# The Electric Field Responses of Inorganic Ionogels and Poly(ionic liquid)s

**DOI:** 10.3390/molecules25194547

**Published:** 2020-10-04

**Authors:** Zhenjie Zhao, Guangchen Zhang, Yuting Yin, Chenjie Dong, Ying Dan Liu

**Affiliations:** State Key Lab of Metastable Materials Science and Technology, and Collage of Materials Science and Engineering, Yanshan University, Qinhuangdao 066004, China; zhenjie_shine@163.com (Z.Z.); 15029276177@163.com (G.Z.); yinyuting34@163.com (Y.Y.); dongchenjie123456@163.com (C.D.)

**Keywords:** ionic liquid, poly (ionic liquid), ionogel, electrorheological fluid, dielectric spectra

## Abstract

Ionic liquids (ILs) are a class of pure ions with melting points lower than 100 °C. They are getting more and more attention because of their high thermal stability, high ionic conductivity and dielectric properties. The unique dielectric properties aroused by the ion motion of ILs makes ILs-contained inorganics or organics responsive to electric field and have great application potential in smart electrorheological (ER) fluids which can be used as the electro-mechanical interface in engineering devices. In this review, we summarized the recent work of various kinds of ILs-contained inorganic ionogels and poly(ionic liquid)s (PILs) as ER materials including their synthesis methods, ER responses and dielectric analysis. The aim of this work is to highlight the advantage of ILs in the synthesis of dielectric materials and their effects in improving ER responses of the materials in a wide temperature range. It is expected to provide valuable suggestions for the development of ILs-contained inorganics and PILs as electric field responsive materials.

## 1. Introduction

**Figure 1:**
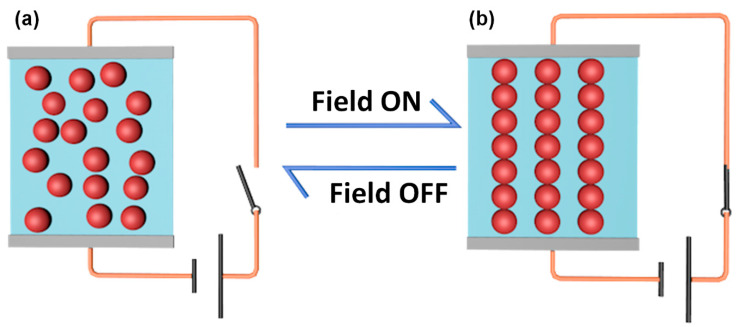
The arrangement structure of the dispersed particles in an ER fluid before (**a**) and after (**b**) the application of an external electric field.

Stimuli-responsive materials are the materials that are able to sense and response to external stimuli, such as temperature, pH value, light, electric and magnetic field, by changing properties or structures in a controllable way [1,2,3,4,5]. For example, light-responsive molecules and temperature-responsive polymers are well known branches of stimuli-responsive materials [6,7,8,9]. Among various stimuli-responsive materials, electrorheological (ER) fluids are considerably attractive because of their rapid and reversible response to electric field. ER fluids are usually suspensions composed of dielectric particles from nanometers to microns dispersed in insulating liquids [10,11]. Without the stimuli of an electric field, the particles dispersed randomly that the suspensions perform like Newtonian fluids. However, they will be like Bingham fluids when an electric field is applied. This is because the particles are able to form chain or column structures in the direction of electric field (Figure 1) [12,13,14]. The electric field controlled liquid-like to solid-like mutual transition makes ER fluids attractive in many engineering applications, such as dampers, shock absorbers, finishing devices, brakes and tactile interfaces [15,16,17,18,19,20].

Properties of ER fluids are closely related to both solid particles and carrier liquids. Comparing with carrier liquids, solid particle materials are more concerned because of their diversity and designability of structures. Therefore, ER materials mainly refer to the solid component of ER suspensions. In the past several decades, researchers paid much attention to designing and developing solid particles with higher sensitivity to electric field and higher ER effect. In the early stage, hydrophilic and porous particles, such as silica, zeolite and titania are mostly used as the dispersed phase of ER fluids. The structures of these particles are easy to absorb water forming hydrous ER materials, which makes the ER effect of these materials depend on the water content absorbed in particles. On the one hand, the polar molecule, H_2_O, contributes to the ER effect of the solid particles. On the other hand, water molecules are easy to evaporate at high temperature which reduces the operating temperature of ER fluids [21]. Then, Block et al. reported semi-conducting polymers as anhydrous ER materials [22,23], which overcame the negative effect of water. After that, anhydrous ER materials won great interest from researchers and diverse kinds of anhydrous ER materials were developed, such as biopolymers, dielectric inorganics and hydrophobic polyelectrolytes [24,25,26,27]. Not only pure materials were studied, but also dual- or multi-component composite materials attracted considerable attention owing to the combined advantages of each component. The development of giant ER fluids by Wen et al. has been a great progress that it broke the upper limit of yield stress predicted in traditional ER fluids [28]. Because of the contribution of polar molecules and high concentration, giant ER fluids are able to exhibit much higher yield stress: tens of kPa or even hundreds of kPa [29,30,31]. However, high yield stress is not the only demand for industrial applications. Low zero field viscosity and long-term stability are also required, obviously which need further more research in the giant ER fluids. Therefore, it is still on the way to develop ER materials or ER fluids with high yield stress, low zero field viscosity, long term stability and wide operating temperature range.

**Figure 2:**
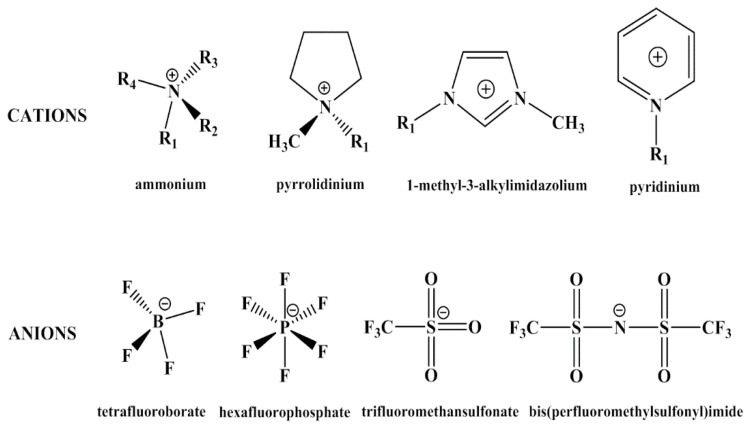
Chemical structures of cations and anions of some ILs.

Recently, ionic liquids (ILs) have been introduced in designing ER materials, creating a new approach to enhance the properties of ER fluids. ILs are a class of organic salts with melting points below 100 °C [32,33]. They usually consist of organic cations and inorganic or organic anions, the unique structures of which give ILs special physicochemical properties, such as low vapor pressure, high thermal stability, high dielectric constant and excellent electrochemical properties [34]. Some of the cations and anions of ILs are shown in Figure 2. Owing to the specific characteristics of ILs, they have been applied in variety of fields including gas absorption [35], catalysis [36,37,38], solar cells [39,40] and organic synthesis [41,42]. In materials synthesis, ILs have been used as not only solvents, but also reactants or templates for achieving inorganics with novel morphologies, structures and improved properties [43]. Sometimes, ILs are immobilized in inorganic frameworks to keep their properties in solids, which are called inorganic ionogels [44]. From this point of view, the matrix of ionogels also could be organics or hybrid materials. Poly(ionic liquid)s (PILs) that refer to a subclass of polyelectrolytes, can be considered one category of ionogels synthesized by polymerizing IL monomers. As a new hybrid material, ionogels considerably enlarge the application of ILs to solid electrolytes, drug delivery and sensors [44]. In recent study, both inorganic ionogels and PILs have been used as ER materials. Marins et al. reported ILs-assisted silica particles which presented higher permittivity and better ER effect than pure silica particles [45,46,47]. Yin et al. have done a series of work on designing PILs-based ER materials with various chemical structures in cations and anions [48,49,50]. Even though ILs have shown excellent physicochemical properties in other fields, there are still several key issues to be solved when applied as ER materials. For example, the counter ions of ILs are mobile at a certain electric field and the mobility of which is closely related to temperature. The result is that at higher temperature electric leakage usually occurs, which is more common in PILs-based ER fluids because of the low glass transition temperature of PILs and the plasticization of organic counter ions.

In this review, we mainly focus on the recent progress of ionogel-based ER materials, including their synthesis methods, ER characteristics and dielectric properties. The ILs-assisted synthesis not only modifies the chemical content of the matrix but also influences the physical properties of the colloidal particles, both of which will obviously affect the response of these particles under electric field [51]. The aim of this work is to highlight the advantage of ILs in synthesis of dielectric materials and their effect in improving the ER responses in a wide temperature range. It should be noted that there are still significant issues explored in the study of this kind of new ER materials. Possible solutions are also suggested to overcoming the problems in the present and further study.

## 2. ILs-Contained Inorganics

The tunability of anions and cations of ILs has shown great potential in the synthesis of materials, which can be used as a solvent and structure directing agent to improve the crystal form and morphology of inorganic materials in the synthesis of inorganic materials [52,53,54]. In addition, in the synthesis of organic materials, ILs can dissolve cellulose [55] and participate in organic synthesis [41,56] by taking advantage of its miscibility with organic matter and high solvation ability. As a green solvent and new type of reaction medium instead of traditional solvents, ILs are very easy to dissolve versatile precursors. It can be fixed or encapsulated in inorganic particles such as silica [57,58], titanium dioxide [59,60], carbon nanotubes [61] and so on, to generate inorganic ionogels [62,63]. Sol-gel processing is the mostly applied method in synthesizing inorganic ionogels. Herein, some examples of ILs-contained or ILs-tethered inorganics will be introduced. These candidates applied as electric field responsive materials will be highlighted.

### 2.1. SiO_2_-Based Ionogels

SiO_2_-based ionogels can be obtained either by dispersing SiO_2_ particles in ILs or adding ILs in the sol-gel process. A large number of studies have shown that nanoparticles are relatively high stable in ILs [64]. Shimano et al. [65] mechanically milled silica and 1-butyl-3-methylimidazole bis(trifluoromethylsulfonyl)imide ([BMIm][TFSI]) to obtain a solid electrolyte with a gel structure. Compared with liquid electrolytes, the high stability of ILs makes gel electrolytes show higher thermal stability, which makes the ionic gels have a wider working temperature range in lithium batteries [66,67]. In addition, within a wide temperature range, ionic gel electrolytes with liquid-like ion mobility may play an important role in electrochemical devices.

**Figure 3:**
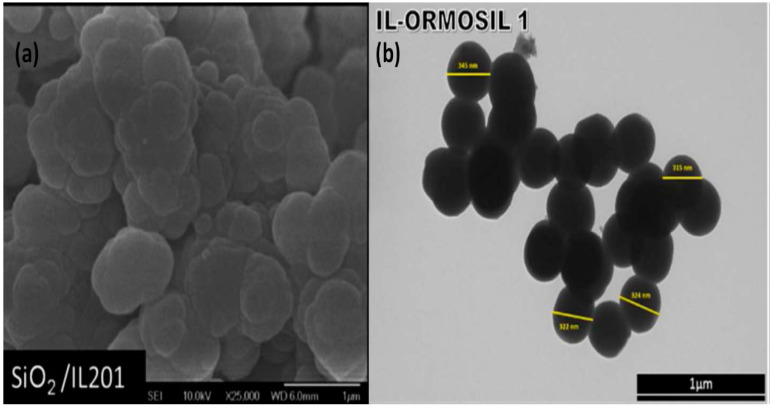
(**a**) SEM image of SiO_2_-based ionogels (SiO_2_/IL201), reprinted with permission from [46]. Copyright RSC, 2011. (**b**) TEM image of SiO_2_-based ionogels (IL-ORMOSIL 1), reprinted with permission from [47]. Copyright Elsevier, 2017.

In the sol-gel preparation of inorganic materials, ILs can be used as solvents to participate in the synthesis of materials. Dai et al. [68] synthesized SiO_2_ aerogel by using 1-ethyl-3-methylimidazolium bis(trifluoromethylsulfonyl)amide ([EMIm][NTf_2_]) as the reaction solvent. On the one hand, the lower vapor pressure of the ILs can prevent the solvent from volatilizing and avoid the collapse of the gel pores caused by the solvent volatilization during the long-term aging process. On the other hand, the ILs are confined in the spatial network of silica, which can improve the stability of the aerogel. The new type of aerogel is expected to be applied as catalyst and insulating material. ILs also can be designed in the form of siliane coupling agent which finally produces a layer of ILs on the surface of SiO_2_ particles by hydrolysis reaction. Marins et al. [45,46,47] synthesized two kinds of SiO_2_-based ionogels by adding pristine ILs and silanized ILs in the sol-gel process. It is found that bare SiO_2_ tends to form a tightly packed structure in the presence of ILs [57], which means that the modification by ILs not only changes the chemical structure but also influences the morphology of the colloidal particles. As shown in Figure 3a, the addition of pristine IL (tri(*n*-butyl)-(tetradecyl)phosphonium dodecylbenzenesulfonate, IL201) during the sol-gel synthesis results in particles with aggregates, suggesting a strong influence of ILs on the morphology of the silica particles. In Figure 3b, silylated imidazolium-based IL is added in the later process of sol-gel synthesis to prepare organically modified silica particles (IL-ORMOSIL 1), which results in spherical particles with organic ILs on the surface.

### 2.2. TiO_2_-Based Ionogels

Because it acts not only as electrode material for lithium batteries but also as matrix for ionogel electrolytes, TiO_2_ is the most popular rechargeable battery material with high capacity [69]. Inorganic matrices that restrict ILs through physical effects usually have a unique mesoporous structure. TiO_2_ inorganic particles prepared by the sol-gel method are rich in 3D network structure. In the hydrolysis-condensation process forming the TiO_2_ inorganic matrix, a large amount of ILs is added. The wormhole nanostructure formed by co-condensation in TiO_2_ is used to confine partial ILs to the inside of the inorganic particles to form a TiO_2_-based inorganic ionogel. The first TiO_2_-based ionogels was synthesized by ultrasonic treatment with the mixture of inorganic titanium salts, methanol, FA, and [BMIm][TFSI] at room temperature [59]. Among many titanium precursors required for the formation of TiO_2_ by the sol-gel method, titanium alkoxide (TBOT) can be used to prepare halogen-free TiO_2_-based ionic gels [66,70]. The porous TiO_2_ matrix can provide ion transport channels and the high conductivity of ILs. Therefore, the electrical conductivity of the TiO_2_-based inorganic ionogel is greatly improved. This is also the reason that TiO_2_-based electrolyte has higher capacity and cycle times, which greatly optimizes the service life of the battery. Besides, the inorganic ionogels obtained by physical limitation has poor long-term stability. In order to compensate for this defect, functionalized ILs can be bonded to the TiO_2_ matrix by stable chemical bonds [71].

### 2.3. Other Inorganics-Based Ionogels

Due to their weakly coordinated molecular ions and high temperature stability, ILs greatly promote the synthesis of materials [72,73]. Here, the following is the application of ILs in metal-organic frameworks (MOFs) and carbon nanotubes (CNTs). MOFs are porous materials constructed from a variety of molecular complexes, which have good mechanical strength and adjustable host-guest interactions. At present, MOFs are widely used in gas storage and phase catalysis and as a new material, they can be used to confine ILs [74,75,76]. Under the condition of ionic thermal method, Ji et al. [77] synthesized a new type of 3D ferroelectric MOFs by using 1-ethyl-3-methylimidazolium bromide ([EMIm]Br) as a solvent and structure-directing agent. The asymmetric structure of the cation [EMIm]^+^, is the key factor in the formation of this helical chain, which further leads to a non-centrosymmetric polar stacking structure. The synthesis of ferroelectric MOF with a higher dielectric constant by ILs will be of great significance for further research on ferroelectric MOFs and general technical applications.

CNTs are usually assembled by tightly entangled graphene sheets with sp^2^ hybrid carbon atoms arranged in a hexagonal shape [78]. Although this structure provides CNTs with unique mechanical and electrical properties, it makes them difficult to dissolve and process [79,80]. By milling CNTs with 1-butyl-3methylimidazolium tetrafluoroborate ([BMIm][BF_4_]) to form a gel, Fukushima et al. [81] found that the cations of ILs will gather on the surface of nanotubes and separate the entangled nanotubes into smaller bundles. The bundles form CNTs gel through physical action. The large surface area of CNTs and the wider electrochemical window of ILs [82] expand the application of this material as electrodes [83].

## 3. Poly(Ionic Liquid)s and Their Composites

ILs can form organic ionic gels with low molecular weight adhesives or polymers [44,84]. As organic molecules, the low molecular weight adhesives are added to the liquids at high temperature and form physical gel when being cooled. When combining the ILs and the polymer to form a specific polymer or composite, they will be characterized with the high conductivity of the ILs and the mechanical properties of the polymer, which are widely used as an electrochemical device material [85,86,87]. Different from hydrophilic inorganic ionogels, the organic ones are hydrophobic that are able to avoid the negative effect of absorbed water of ER materials.

### 3.1. Poly(Ionic Liquid)s

**Figure 4:**
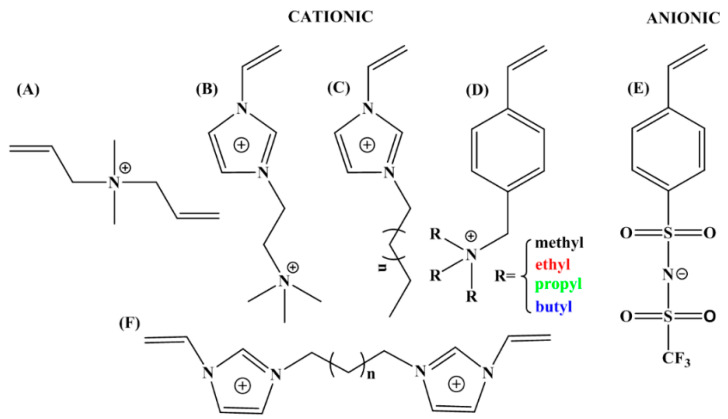
Chemical structures of cationic and anionic IL monomers for PILs: (**A**) [DADMA]^+^; (**B**) [VIm]^+^ [BETA]^+^; (**C**) [CnVIm]^+^; (**D**) [VBTRA]^+^; (**E**) [STFSI]^−^; (**F**) [CnDVIM]^2+^.

Poly(ionic liquid)s (PILs) that use ILs as monomer and whose anionic or cationic center is restricted to the polymer backbone are a special type of polyelectrolyte [88]. The methods to prepare PILs include free radical polymerization, controlled polymerization and so on. From monomer to polymer, some special properties of ILs (such as negligible vapor pressure, thermal stability and wide electrochemical stability window) are transferred to the PILs chain and the diversity of ILs will enrich the characteristics and applications of PILs, which attract widespread attention in the study of polymers and other materials [32,89]. In practical applications, PILs always exhibit relative low conductivity because of the restriction effect of polymer backbone to mobile ions of the IL side groups. It is necessary to add highly conductive lithium salts to prepare electrolyte materials [34]. PILs are not only good candidates for electrolytes in batteries but also display stimuli-responsive properties, for example, the sensitivity to CO_2_ [90]. Interestingly, the low conductivity of PILs is just suitable for the application of electro-responsive ER materials, which makes PILs are attractive in the field of stimuli-responsive materials. Figure 4 shows the chemical structures of IL monomers and crosslinkers for synthesizing PILs that have been applied as ER materials.

**Figure 5:**
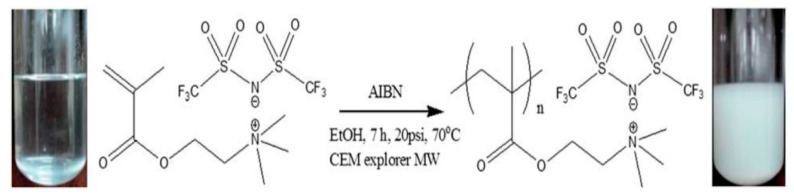
The microwave-assisted synthesis process of P[MTMA][TFSI], reprinted with permission from [91]. Copyright RSC, 2014.

**Figure 6:**
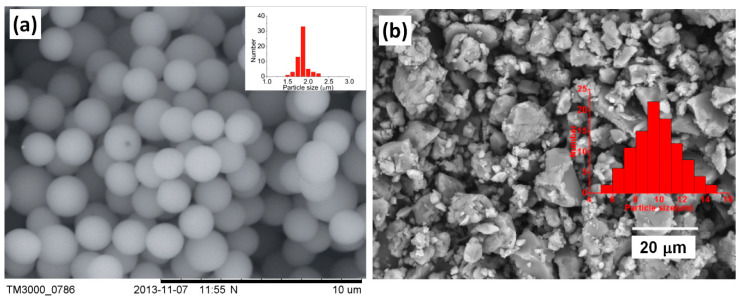
(**a**) SEM image of P[MTMA][TFSI] particles, reprinted with permission from [91]. Copyright RSC, 2014. (**b**) SEM image of P[VIm][TFSI] particles, reprinted with permission from [92]. Copyright Elsevier, 2019.

**Figure 7:**
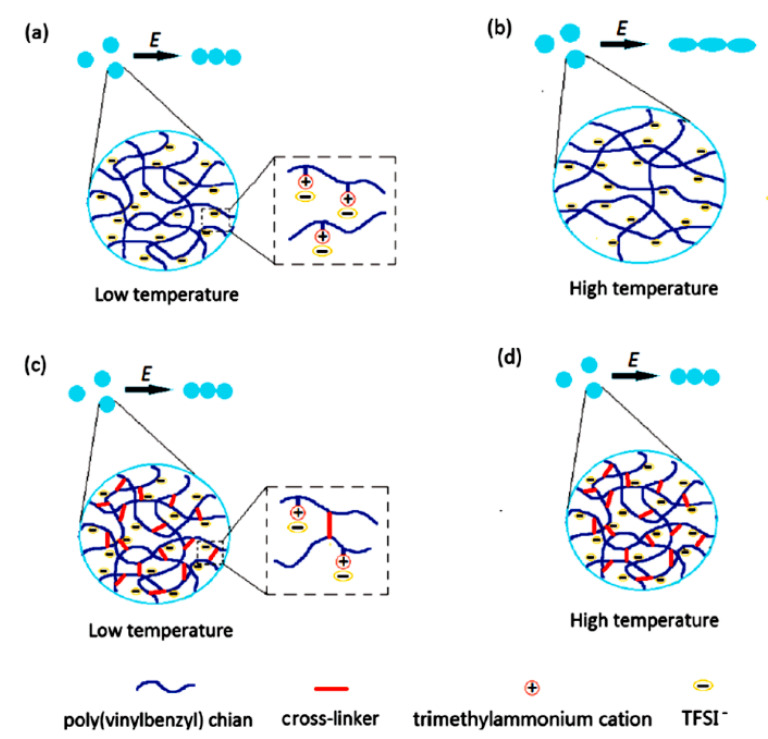
Schematic diagram of chain formation of uncross-linked (**a**,**b**) and cross-linked (**c**,**d**) PILs particles at low and high temperatures, reprinted with permission from [93]. Copyright RSC, 2017.

When used as ER materials, PILs should be in the solid colloidal state with particle size ranging from submicrons to microns. A series of PILs with different chemical structures were prepared using either dispersion or solution polymerization method for this purpose [48,91]. Figure 5 shows a certain process of the microwave-assisted synthesis of poly[2-(methacryloyloxy)ethyltrimethylammonium bis(trifluoromethane sulfonylimide)] (P[MTMA][TFSI]). The reaction system transforms from transparent to opaque white because of the growth of P[MTMA][TFSI] particles. It has confirmed that the synthesis methods influences the morphology of the PILs particles significantly. As shown in Figure 6a, the spherical particles with an average size of 1.8 μm are prepared by the microwave-assisted dispersion polymerization method [91]. Figure 6b are the poly [(vinyl imidazole) bis(trifluoromethane sulfonylimide)] (P[VIm][TFSI]) particles prepared by a solution polymerization process using DMF as the solvent [92]. The particles from this process are irregular in shape and with much larger average size (~10 μm). Sometimes diethyl ether is used to make PILs precipitate in the original solvent (e.g., DMF). These precipitated PILs particles are also irregular in shape rather than spherical. In order to improve the thermal stability and mechanical properties of PILs, crosslinking agent is added in the polymerization process. It is also possible to prepare self-crosslinked PILs using bisvinylimidazolium-based monomers (Figure 4F). By varying the amount of crosslinking agent or the length of alkyl spacer, the mesh size of crosslinking network can be adjusted, which significantly influences the movement of backbones and counter ions of PILs. As shown in Figure 7, Liu et al. [93] prepared cross-linked PILs, poly[p-vinylbenzyltrimethylammonium bis(trifluoromethane sulfonimide)] (P[VBTMA][TFSI]). Comparing with the uncross-linked one, which shows enlarged free volume at higher temperature because of the uncoiling of polymer chain, the cross-linked P[VBTMA][TFSI] provides smaller cavities for ion transport at both room and elevated temperature.

### 3.2. Composites of P(Ionic Liquid)s

**Figure 8:**
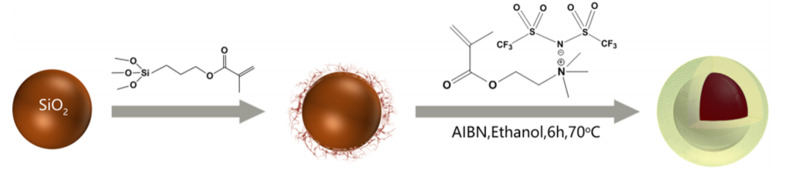
Schematic preparation of SiO_2_@P[MTMA][TFSI] core-shell particles, reprinted with permission from [95]. Copyright ACS, 2018.

As introduced above, ion transport ability in uncross-linked PILs is accelerated at temperature higher than glass transition temperature (*T*_g_), which could result in large current leakage and then deteriorating ER effect. Therefore, one of the issues for PILs-based ER materials is to improve their thermal stability or broaden their working temperature range. Introducing inorganic component to PILs to prepare PILs-inorganic composite is an effective method to improve the thermal stability of PILs. Both general composites and core-shell structured composites were prepared for this purpose. When a polymer is prepared on a inorganic substrate surface (e.g., SiO_2_), the strong interfacial interaction between the polymer and the inorganics will affect the *T*_g_ of the polymer by changing the stereoregularity of the polymer [94]. That is why core-shell particles with a SiO_2_ core and PIL shell were designed for the above issue [95]. Figure 8 is the schematic preparation process of SiO_2_@P[MTMA][TFSI] core-shell particles. In the first step, the surface of SiO_2_ particles is grafted by coupling agent (3-methacryloxypropyltrimethoxy-silane, MPS) via the hydrolyzation reaction. Then in the second step, IL monomer ([MTMA][TFSI]) is added and polymerized on the surface of SiO_2_ particles. Another way to prepare PILs-contained core-shell particles is the Pickering emulsion polymerization method [96,97,98,99,100]. In this process, inorganic nanoparticles rather than organic molecules are applied as emulsifier, producing inorganic nanoparticles-wrapped PILs particles. By using this way, Zhao et al. prepared PILs encapsulated nano-SiO_2_ composite, in which process nano-SiO_2_ was applied as a solid emulsifier [101]. It indicated that the SiO_2_ nanoparticles on the surface of the composite particles can largely decrease the leaking current density and improve the working temperature range of the ER fluid.

As a conductive polymer, polyaniline (PANI) has lower density, better processing properties, adjustable conductive properties, conjugate structure, and a wider potential for chemical functionalization [102]. It has been widely used in sensors, antistatic products as well as ER responsive materials [103,104,105,106,107,108]. Because of the excellent ER properties of PANI, it is applied to prepare composite materials with PILs to overcome the drawbacks of PILs. Zheng et al. [109] prepared PILs-encapsulated PANI composite particles. The synthesis process is a two-step method that the hydrophilic poly(vinylbenzyl)trimethylammonium chloride (P[VBTMA]Cl) is mixed with aniline firstly to obtain the PANI@P[VBTMA]Cl gel by oxidation polymerization and then the hydrophobic PANI@P[VBTMA][PF_6_] composite particles are obtained by ion exchange. The intrinsic conductivity of PANI suppresses the charged surface of P[VBTMA][PF_6_], which reduces the Zeta potential of PANI@P[VBTMA][PF_6_] to a lower level. In addition, core-shell structured PILs/PANI were also prepared using P[MTMA][TFSI] as core and semiconducting PANI as shell to limit the ion leakage of PILs [110]. The successful synthesis is mainly based on the good affinity between the P[MTMA][TFSI] core and the aniline monomer. Thus, interfacial polymerization of aniline could be realized by adding initiator or oxidant to the system. Low temperature polymerization at 0 °C is also required to slowdown the reaction rate of monomers and to avoid homogenous nucleation of aniline monomers.

**Figure 9:**
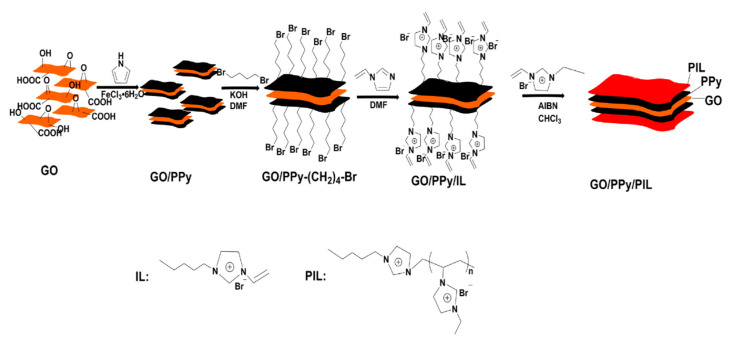
Schematic diagram of fabrication of GO/PPy/PIL nanosheets, reprinted with permission from [115]. Copyright Elsevier, 2018.

Recently, graphene oxide (GO) has been widely used in electronic devices and capacitors due to its unique two-dimensional structure, high specific surface area, good polarizability and low conductivity [111,112]. However, for the application in ER fluids, the conductivity of GO is still higher than that of conventional ER materials, which easily causes electric breakdown in the electric field and reduces the ER effect of GO [27,113]. In order to improve the ER effect of GO, other inorganic substances or polymers are introduced to prepare composite particles [114]. By using a multi-layer composite method, Chen et al. [115] coated a polypyrrole (PPy) layer and a PILs layer on the surface of GO in sequence and obtained a multi-layered composite material with low conductivity and high ER effect. Firstly, the PPy layer is coated by an in situ polymerization process [116]. Then 1, 4-dibromobutan and 1-vinylimidazole are grafted on the surface of GO/PPy in turn and finally a PILs layer is formed by radical polymerization. The schematic synthesis process is shown in Figure 9.

## 4. ER Responses

As it has been introduced in Part 2, ILs and solid network can form ionic gel by restricting IL molecules in the solid network or on the surface of particles. The addition of ILs gives the ionic gel a unique responsiveness to electric field stimuli, which makes the ionic gel a good candidate for ER responsive materials. The high dielectric constant and ionic conductivity of ILs are expected to improve the ER response of the ionic gels. Besides, the ER effect of these materials will closely depend on the structures of both matrix and ILs.

### 4.1. ER Response of Inorganic Ionogels

By physical or chemical methods, ILs are confined in inorganic particles, which will enhance the responsiveness of inorganic particles in an electric field. Among the numerous inorganic ER materials, silica is widely used because of its easy synthesis, low cost and adjustable properties [117]. Although the ER effect of silica is not outstanding, adding a small amount of water or other polar molecules will enhance its ER effect [118,119,120,121]. According to the Stöber procedure, by adding different types of ILs during the hydrolysis of tetraethylorthosilicate (TEOS) [45,46], the ILs and silica can form ionic gel through physical interaction. Under an electric field, the ILs doped inside the ionic gel will produce charge migration, which affects the ion concentration and makes the gel possess a greater dielectric constant and a higher conductivity. The ionic gel shows obvious ER performance, which is several times that of pure silica. At the same time, according to the principle of “similar polarity”, when the anions and cations of the ILs doped physically have long alkyl chains, their wettability with the silicone oil will be increased, which causes partial anions and cations to leak out of the ionic gel. This effect increases the ionic conductivity of the insulating oil, reduces the dielectric constant of the ionic gel and eventually decreases the ER properties.

Compared with physical doping, chemical grafting by covalent bond is more effective in fixing ILs in inorganic matrix. ILs-grafted silica particles not only prevents its electric leakage during use but also provides a higher polarization rate and stronger ER performance [47]. In a word, during the synthesis process of silica, adding ILs can significantly improve the ER response of silica gels. However, other ILs-contained inorganics have not been applied as ER materials. There is still much work to do to explore the electric field responsive properties of inorganic ionogels.

### 4.2. ER Responses of PILs

PILs that are used as a new type of electrolyte material have a macromolecular structure composing of ILs as repeating units and connecting by a polymer backbone [34,85,88]. The formation of PILs reduces the number of movable ions of the system and the network structure limits the movement of chain segments and the long-distance migration of ions, which makes the conductivity of PILs significantly decrease to the range suitable for ER fluids. In ER fluids, proper conductivity helps to produce a stronger ER effect [122]. When PILs are used as the dispersed phase of ER fluids, the high density of anion and cation will generate stronger dielectric polarization, which leads to the enhancement of ER response [91]. In addition, due to the diverse chemical structures of ILs monomers, their ER response is able to be adjusted by modifying the chemical structures of ILs. It also means the chemical structures of cations, anions and even the backbones have significant effect on the ER response of PILs.

#### 4.2.1. Chemical Structure of Anions

**Figure 10:**
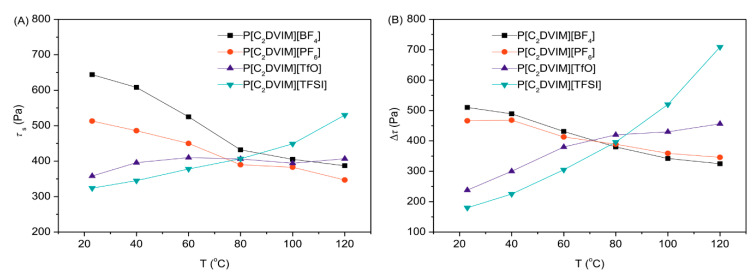
Temperature dependence of static yield stress (*τ*_s_) (**A**) and Δ*τ* (**B**) for P[C_2_DVIM]X ER fluids (*φ* = 20 vol%). The electric field strength is 3 kV/mm and shear rate is 630 s^−1^. Reprinted with permission form [125]. Copyright Elsevier, 2019.

For ILs, the structures and properties of anion can affect the non-electrostatic interaction between ILs [123]. Among the anions of ILs, [TFSI]^−^ is an hydrophobic one, whose large size and weak electrostatic interaction with cations lead to relatively large ion mobility and wide use in electrolytes [48,124]. Whereas it may be not the same in ER fluids. To explore the influence of ion mobility of anion on ER effect, Dong et al. [48,49] studied the ER effect of PILs with the same polymeric cations but different anions. In the characterization of ER performance, dynamic yield stress (*τ*_y_) represents the response strength of suspension particles to the electric field. When comparing the ER responses of these PILs, it is found that these ER fluids have similar zero field viscosities due to the similar particle size of PILs. Under an electric field, the ER responses of the ER fluids of PILs with different anions are different and the order of *τ*_y_ is P[VBTMA][TfO] > P[VBTMA][BF_4_] > P[VBTMA][TFSI] > P[VBTMA][PF_6_]. Since they have the same cation, the different performance of ER response may be related to the anion. It seems that PILs with smaller anion size will exhibit a stronger ER effect. However, the inorganic or organic nature of the counter anions also has effect on the ER performance of PILs, for example, the temperature dependence of their ER performances. The PILs with inorganic anions show decreasing of static yield stress (*τ*_s_) with temperature, on the contrary, increasing of *τ*_s_ with temperature for the PILs with organic anions (Figure 10A). The similar trend is observed for Δ*τ* in Figure 10B. Note that *τ*_s_ used to evaluate the ER effect is the stress that drives the solidified ER fluid to flow and it can be approximately obtained by extrapolating the shear stress curve to zero shear rate. Δ*τ* = *τ*_E_ − *τ*_0_ is the electric field induced increment of shear stress, where *τ*_E_ and *τ*_0_ represent the shear stress measured at applied electric field strength and zero electric field. For the cross-linked PILs, they have reduced free volume for counter anions to transport through. Therefore, mobility of anion is more sensitive to its own size, which was confirmed by Liu et al. [125] via studying the self-crosslinked PILs with different types of anions. They also found that the ER response of inorganic anion was significantly higher than that of organic anion. Inorganic anions usually have smaller size, the easy migration of which generates a greater interfacial polymerization and a stronger ER response. Therefore, it can be concluded that when PILs are used as suspension particles, the anion of PILs that has the relatively small size will lead to stronger ER effect.

**Figure 11:**
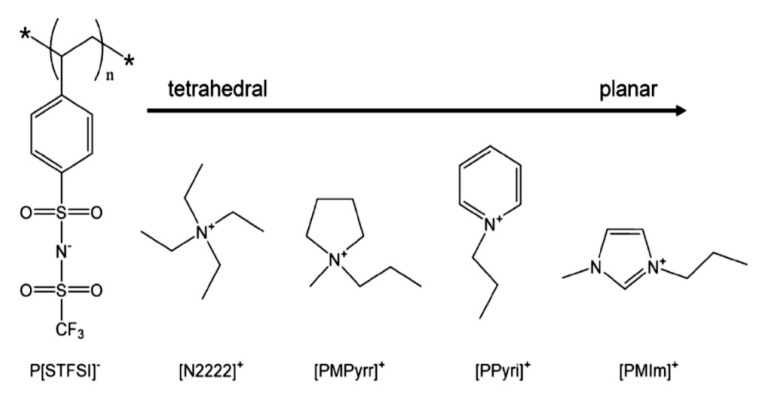
Chemical structure of anionic PILs with different geometry of counter cations, reprinted with permission from [126]. Copyright Elsevier, 2020.

Typical low temperature ER effect is presented by an anionic PILs, which is considerably attractive because good low temperature performances of ER fluids are rarely reported using common ER materials. The poly[4-styrenesulfonyl (trifluoromethylsulfonyl) imide]-based anionic PILs containing counter cations with varied geometries from tetrahedron to plane (Figure 11) showed excellent ER effect with yield stress at 0 °C is about 1500 Pa (*E* = 3 kV/mm, *φ* = 20 vol%) [126]. The geometry of the counter cation has a significant effect on *T*_g_ of the PILs. As it varies from tetrahedron to plane, *T*_g_ of the PILs decreases, resulting in a inverse sequence of ER effect at 0 °C.

#### 4.2.2. Chemical Structure of Cations

The size of cation and the interaction between anions will affect the conductivity of ILs and PILs [127,128]. In order to explore the effect of cations on the ER response of PILs, He et al. [129] synthesized PILs with different tethered cations dimethyldiallylammonium ([DADMA]^+^), benzylethyl trimethylammonium ([VBTMA]^+^) and 1-vinyl 4-ethylimidazolium ([C2VIm]^+^) and the same counter anion hexafluorophosphate (PF_6_^−^). The chemical structures of the cations are shown in Figure 4. Under an external electric field, suspensions of the three PILs show different ER response degrees and the trend of ER effect is P[DADMA][PF_6_] > P[VBTMA][PF_6_] > P[C2VIm][PF_6_]. Under the situation of similar particle morphology and zero field viscosity, the chemical nature of cation or the interaction between anion and cation may be the reason that leads to the different ER responses. The ion-pair interaction energy between [DADMA]^+^ and PF_6_^−^ is the smallest, which results in higher ion mobility, then produces faster interfacial polarization and stronger ER response under an electric field. To compare the effect of ion number density on ER effect, Wang et al. [92] synthesized single-cation and double-cation PILs. Due to the higher ion number density in the dual-cation PILs, it exhibits higher polarizability and stronger ER response under an electric field. In addition, to study the influence of the chemical structure of the counter cation on the ER effect of anionic PILs, Zhao et al. [50,92,126,130] synthesized poly[4-styrenesulfonyl (trifluoromethylsulfonyl) imide (P[STFSI][X]) with anionic polymer backbone and cationic counterion. It is found that the size of cations did not show a monotonic influence on the ER effect of the PILs. Both lower activation energy for ion motion and a relative uniform amorphous structure are necessary for fast interfacial polarization rate and large polarization intensity. It means the ER effect is not only related the chemical structure of cations but also depends on the micromorphology of PILs.

#### 4.2.3. Length of Alkyl Chain

When using molecular dynamics simulation to study the influence of cationic alkyl chain length on the properties of ILs [131,132,133,134], a local area of polar and non-polar will appear as the alkyl chain length increases. The local environment can affect the viscosity and ion conductivity of ILs. In order to understand the influence of side alkyl chain length of the immobile quaterammonium cation on ER effect, Dong et al. [48,135] synthesized PILs with styrene skeleton and quaternary ammonium cation with different lengths of alkyl chain on the counterpoint through free radical polymerization. The chemical structure of the monomers for PILs are shown in Figure 4D. Although the synthesized PILs have the similar particle size, morphology and type of anion and cation, they exhibited various ER responses. As the length of the alkyl chain increases, the volume of the cation becomes larger, which causes the local movement of the counterion to be restricted by the steric effect, thereby reducing the number of movable ions and the ion conductivity of the system. It leads to slower interfacial polarization rate and lower ER response of PILs at electric fields. Similar conclusion is obtained by Zhang et al. [136,137] upon the study of immidazolium-based PILs with different alkyl chain lengths on the immidazolium ring. It is found that as the alkyl chain becomes longer, the ER effect of the PILs becomes weaker. In other words, the shorter alkyl chain on the cation in PILs produces stronger ER performance.

However, when the alkyl chain acts on the cross-linked PILs, the opposite result will appear. In the study of the self-crosslinked PILs, divinylimidazolium monomers with different alkyl spacer lengths were used [138]. In the spatial structure of PILs, as the length of the alkyl spacer increases, the PILs have a larger cross-linked network mesh structure through the endoplasmic reticulum effect. The larger space structure reduces the energy required for ion diffusion and increases the ion mobility so that PILs with a large mesh structure have greater ionic conductivity and stronger ER response.

#### 4.2.4. Crosslinking Structure

It has been demonstrated that due to the plasticizing effect of organic anions (such as [TFSI]^¯^) the *T*_g_ of PILs will decrease. Therefore, as temperature increases, the chain motion of PILs is intensified and the ion migration is promoted by coupling effect. Large leakage current will occur and limit the working temperature range of the PILs-based ER fluids [49,138]. As it has been mentioned above, the crosslink to PILs is used to solve this problem [93]. After cross-linking, the PILs form a high-density cavity and a rigid network, which limit the long-range migration of ions and let counter ions (TFSI¯) only move locally in the cross-linked network. In addition, the rigid structure after cross-linking increases the *T*_g_ of PILs, inhibiting chain movement and decreasing polymer free volume at high temperatures. When the temperature is increased, the cross-linked PILs are not easy to swell to become soft. Through a moderate crosslinking degree, it will slightly reduce the leakage current density and broaden the working temperature range of PILs, without sacrificing the ER effect of PILs significantly.

#### 4.2.5. PILs-Based Composites

As it has been mentioned above, the preparation of PILs-based composite ER materials is to restrict the ion leakage of PILs in ER fluid. Other advantages achieved are the enhanced ER effect and broad working temperature range as well. Lei et al. [95] chose SiO_2_ particles as hard core and prepared SiO_2_@P[MTMA][TFSI] particles with core-shell structure. Because of the strong interaction between PILs shell and SiO_2_ core, the segmented movement of PILs is limited, leading to the increase in *T*_g_ of the PILs. The core-shell particles SiO_2_@ P[MTMA][TFSI] still have a strong ER response when the temperature is up to 120 °C. In addition, the SiO_2_@P[MTMA][TFSI] particles provide two interfaces: the interface between SiO_2_ core and the PILs shell where counter ions will gather, and the interface between the PIL shell and silicone oil. The core-shell structure of two interfaces is beneficial to increase the interfacial polarization, which makes SiO_2_@PILs have strong ER performance under the electric field. For the PILs encapsulated nano-SiO_2_ (PILs/SiO_2_) particles by Picking emulsion polymerization [101], nano-SiO_2_ on the surface of the composite particles seems like a cross-linking point which will increase the activation energy of ion migration of PILs and significantly inhibit the leakage current at high temperature. As a result, the leakage current of composite particles is small at 100 °C. Compared with pure PILs, PILs/SiO_2_ has outstanding temperature stability and stronger ER effect. Therefore, the introduction of inorganic SiO_2_ into the PILs can significantly reduce the leakage current density and improve the temperature stability, which provides a new idea for developing ER fluids suitable for in high temperature environments.

In order to overcome the charged nature of the PILs surface after ion exchange, PANI is used as either a filler or a shell material to prepare PANI filled PILs or PANI coated PILs (PANI@PILs) [109,110]. The change of surface potential makes the composites behave stronger ER response under the electric field and the yield stress obtained under an electric field of 4 kV/mm is about 2.3 kPa, which is twice that of pure PILs and 2.5 times that of PANI. In order to study the effect of reducing ion leakage and the degree of polarization matching between PANI and PILs, PANI was treated by alkali to obtain different forms [110,139]. After PANI undergoing different treatments, PILs/PANI (NH_3_) particles exhibit a stable shear range at room temperature, while PILs/PANI (N_2_H_4_) particles have stable shear at high temperatures. These differences are because PANI treated with ammonia and hydrazine have different dielectric polarization ratios. When PANI and PILs have similar dielectric matching, they will exhibit stable shear flow curves. Therefore, PANI@PILs can exhibit a strong ER response by changing the charge state of the particle surface. The coating of PANI on the surface of PILs effectively inhibits the positively charged surface state and produces a stronger ER effect.

## 5. Dielectric Analysis

It is generally accepted that the response of ER materials to the electric field is from the interfacial polarization of dispersed particles [140] and the dielectric properties of particles affect the polarization strength which will affect ER performance of the particle suspensions [141,142]. In the research of ER fluids, to analyze the rheological behavior of ER fluids, the dielectric constant and relaxation time are two important parameters [113]. ER fluids with good performance should have a large dielectric relaxation strength and a suitable dielectric relaxation peak in the frequency range of 10^2^–10^5^ Hz [143]. Dielectric spectrum analysis method has advantages such as broad measurement frequency, non-invasive and rapid measurement. Nowadays, more and more ER researches are using dielectric analysis method to understand the mechanism of the ER phenomena.

**Figure 12:**
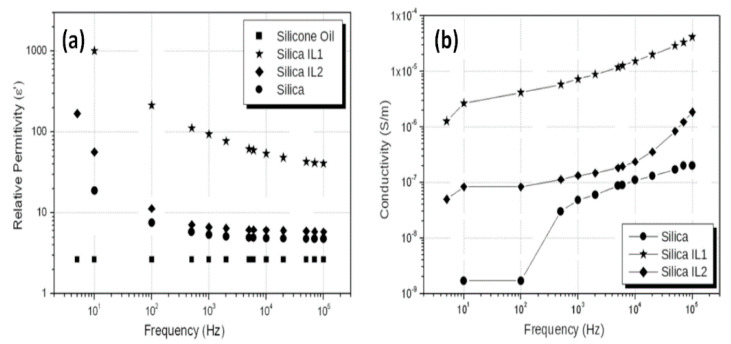
Dependence of relative permittivity (**a**) and conductivity (**b**) on frequency for the silica, silica/IL1, and silica/IL2 suspensions, reprinted with permission from [45]. Copyright Elsevier, 2013.

In the study of silica-based ionogels, which were synthesized by adding 11-carboxyundecyltriphenylphosphonium bromide (IL1) and octadecyltriphenylphosphonium iodide (IL2) in the sol-gel process, it is found that both relative dielectric constant (Figure 12a) and conductivity (Figure 12b) of silica/ILs are higher than pure silica [46], corresponding to the better ER response than that of pure silica as well. Significantly, among the three samples, the silica/IL1 exhibits the highest relative permittivity (~1100 at 10 Hz). It would be related the chemical structure of IL1 which contains a carboxylic acid tail in the triphenylphosphonium cation. Similarly, ILs organically modified silica particle also exhibits higher ER response and higher dielectric properties [47].

To fit the dielectric data of ER materials, a relaxation Equation, which includes a Cole-Cole’s term, a DC conductivity term and an electrode polarization (EP) term, was used to analyze the dielectric spectra [139]:

(1) $$\varepsilon^{*}\left(\omega \right)=\varepsilon^{\prime }+{i\varepsilon }^{\prime \prime }=\varepsilon_{\infty }^{\prime }+\frac{\Delta \varepsilon^{\prime }}{1+{\left(i\omega \lambda \right)}^{\beta }}+i\frac{\sigma }{\varepsilon_{0}\omega }+A\omega^{-n}$$

where $\Delta \varepsilon^{\prime }=\varepsilon_{0}^{\prime }-\varepsilon_{\infty }^{\prime }$ is the dielectric strength ($\varepsilon_{0}^{\prime }$ and $\varepsilon_{\infty }^{\prime }$ are the limit values of the relative permittivity at the frequencies below and above the relaxation frequencies, respectively), *λ* = 1/*ω*_max_ the relaxation time, *ω*_max_ is the local angular frequency of dielectric loss peak, *β* is the Cole-Cole parameter indicating the distribution of the relaxation time, *σ* is the DC conductivity, *n* is related to the slope of EP’s high frequency tail, and *A* is related to the amplitude of EP.

If the dispersed phase of ER fluid consists of two components, two Cole-Cole’s terms can be used to analyze the dielectric properties. The equations of *ε*′ and *ε*″ are shown below, where subscript 1 and 2 are for each component respectively [139].

(2) $${\varepsilon^{\prime }=\varepsilon }_{\infty }^{\prime }+\Delta \varepsilon_{1}^{\prime }\left(\frac{1+{\left({\omega \lambda }_{1}\right)}^{\beta_{1}}\cos\left(\frac{{\pi \beta }_{1}}{2}\right)}{1+2{\left({\omega \lambda }_{1}\right)}^{\beta_{1}}\cos\left(\frac{{\pi \beta }_{1}}{2}\right)+{\left({\omega \lambda }_{1}\right)}^{{2\beta }_{1}}}\right)+\Delta \varepsilon_{2}^{\prime }\left(\frac{1+{\left({\omega \lambda }_{2}\right)}^{\beta_{2}}\cos\left(\frac{{\pi \beta }_{2}}{2}\right)}{1+2{\left({\omega \lambda }_{2}\right)}^{\beta_{2}}\cos\left(\frac{{\pi \beta }_{2}}{2}\right)+{\left({\omega \lambda }_{2}\right)}^{{2\beta }_{2}}}\right){+A\omega }^{-n}$$

(3) $$\varepsilon^{\prime \prime }=\Delta \varepsilon_{1}^{\prime }\left(\frac{{\left(\omega \lambda_{1}\right)}^{\beta_{1}}\sin\left(\frac{\pi \beta_{1}}{2}\right)}{1+2{\left(\omega \lambda_{1}\right)}^{\beta_{1}}\cos\left(\frac{\pi \beta_{1}}{2}\right)+{\left(\omega \lambda_{1}\right)}^{2\beta_{1}}}\right)+\Delta \varepsilon_{2}^{\prime }\left(\frac{{\left(\omega \lambda_{2}\right)}^{\beta_{2}}\sin\left(\frac{\pi \beta_{2}}{2}\right)}{1+2{\left(\omega \lambda_{2}\right)}^{\beta_{2}}\cos\left(\frac{\pi \beta_{2}}{2}\right)+{\left(\omega \lambda_{2}\right)}^{2\beta_{2}}}\right)+\frac{\sigma }{\varepsilon_{0}\omega }$$

**Figure 13:**
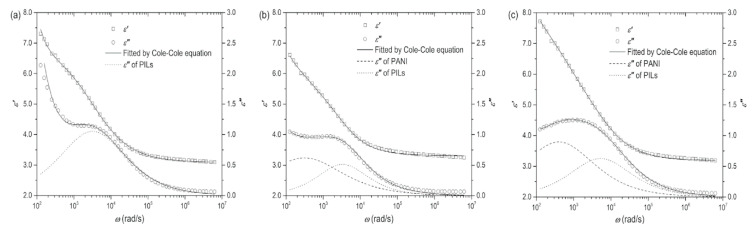
Dielectric spectra of the suspensions of P[MTMA][TFSI] microspheres (**a**) P[MTMA][TFSI]@PANI (thin) microspheres (**b**) and P[MTMA][TFSI]@PANI (thick) microspheres (**c**) (*φ* = 20 vol%, T = 23 °C), reprinted with permission from [110]. Copyright John Wiley & Sons, 2018.

The above Equations are suitable for the ER systems containing composite as dispersed phase, such as the core-shell particles. Compared with hydrous ER fluid, the anhydrous PILs-based ER fluid has incomparable advantages, but it also faces the irreversible leakage of mobile ions from particles into carrier liquid under high electric field, which will increase energy consumption. In order to solve this problem, Zheng et al. [110] coated a layer of PANI on the surface of PILs particles, which successfully limits the ion leakage, reduces the energy consumption and enhances the interface polarization due to the more charge accumulation at the interface between PANI and PILs. Figure 13 shows the dielectric spectra of the ER fluids based on P[MTMA][TFSI], P[MTMA][TFSI]@PANI (thin) and P[MTMA][TFSI]@PANI (thick) paritcles. In Figure 13a, it is observed that both *ε*′ and *ε*″ increase rapidly in the low frequency regime, which is caused by the high DC conductivity of of the PILs and the electrode polarization. When the PILs particles are coated by PANI, it is found in Figure 13b,c that the rapid increase in both *ε*′ and *ε*″ at the low frequency regime is suppressed, indicating the critical effect of PANI in isolating the ion leakage of the PILs core. It is possible to obtain dielectric relaxation information of each component (P[MTMA][TFSI] and PANI) by using Equaitons (2) and (3) to fit the dielectric spectra of the P[MTMA][TFSI]@PANI-based ER fluids. Two relaxation process are observed: one is for the P[MTMA][TFSI] core and the other is for the PANI shell.

Moreover, the plasticizing effect of polyatomic fluorinated counter ions ([TFSI]¯) reduces the *T*_g_ of PILs, which decreases the thermal stability of the PILs-based ER fluids. In order to improve the thermal stability of the ER fluids, PILs encapsulated nano-SiO_2_ composite particles and SiO_2_@P[MTMA][TFSI] core-shell particles were designed and synthesized [95,101], which limit the movement of chain segments and the ionic transport of PILs. After analyzing dielectric properties of the SiO_2_@P[MTMA][TFSI] core-shell particles, it is found that the hard SiO_2_ core not only increases the dielectric strength of the core-shell particles but also restrains the segment relaxation of the P[MTMA][TFSI] shell. As shown in Table 1, the SiO_2_@P[MTMA][TFSI] ER fluid exhibits higher value of Δ*ε*′ but longer relaxation time (*λ*) due to the effect of hard core. Chemical crosslinking to PILs is also an effective way to broaden the working temperature of PILs-base ER fluid by restricting the segment movement of PILs with cross-linked networks. However, these networks also provide mobile counter ions with smaller cavities which decrease the mobility of the ions, resulting in an obvious increase in relaxation time.

By studying the dielectric spectra of the ER materials at different temperatures, it is found that the dielectric relaxation of the ER fluids moves to high frequency region due to the faster relaxation at higher temperature. In addition, both *ε*’ and *ε*” rise up to higher value in the low frequency region because of the electrode polarization. It is also noted that for the composite particles, the relaxation peak is broader than that shown by the single component particles, resulted by the two relaxation processes of the composite. It means temperature affects the migration of ions in ILs or PILs which is related to the relaxation time. It is found that the reciprocal of the dielectric relaxation time (*λ*^−1^) follows the Arrhenius equation [139]:

(4) $$\lambda^{-1}\propto \;e^{-E_{a}/RT}$$

where *E*_a_ is the activation energy, *R* is the molar gas constant, and *T* is the absolute temperature. Upon the study of the Arrhenius dependence of the PILs-based ER fluids, it is indicated that the ion motions in PILs or its composites are from the thermal diffusion, and not coupled with the chain segment below *T*_g_ of the PILs. The situation is much different when temperature is higher than *T*_g_, the activation of segment relaxation drives *λ*^−1^ to depart from the Arrhenius line [95].

**Figure 14:**
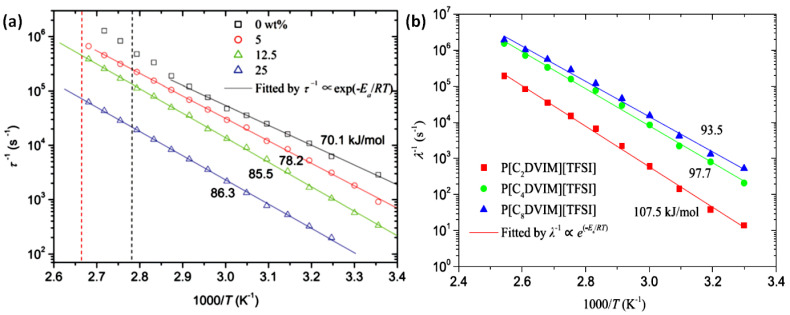
(**a**) Temperature dependence of the relaxation time of the ER fluids based on of P[VBTMA][TFSI] and cross-linked P[VBTMA][TFSI] particles with different weight ratios of the cross-linker (**a**), reprinted with permission from [93]. Copyright RSC, 2017. (**b**) The self-crosslinked PILs with different alkyl space length, reprinted with permission from [138]. Copyright Elsevier 2019. The solid lines in (**a**,**b**) are fitted by the Arrhenius equation.

The activation energy *E*_a_ is actually the potential energy barrier for ion transport, which is closely related to the chemical and physical structure of PILs. For the core-shell particles, for example the P[MTMA][TFSI]@PANI particles, they showed higher *E*_a_ than pure P[MTMA][TFSI]. Chemical crosslinking to PILs rise the *E*_a_ to higher value as well [93]. With the increase of crosslinking degree, both the relaxation time and *E*_a_ of the ER fluid increased. Figure 14a shows the temperature dependence of the relaxation time (*τ*) of the cross-linked PILs, in which the slope of the fitting line is *E*_a_. By comparing the slopes of different PILs, it is found that the higher the crosslinking degree of PILs is, the greater the *E*_a_ is. It means the crosslinking hinders the ion migration, which increases the *E*_a_ and prolongs the relaxation time. Another way to adjust the crosslinking degree or mesh size of the crosslinking network of PILs is to apply cross-linkers with different length of alkyl spacer. As shown in Figure 14b, as the alkyl spacer becomes longer, the *E*_a_ of the ER fluid decreases from 107.5 to 93.5 kJ/mol. In addition, comparing the ER response and dielectric spectra of the suspensions of P[MTMA][TFSI] and SiO_2_@P[MTMA][TFSI], we found that the hard SiO_2_ core can influence the ion dynamics, restrain the segment softening of P[MTMA][TFSI] shell and increase the interfacial polarization strength of SiO_2_@P[MTMA]-[TFSI]-based ER fluid by substrate confinement effect.

## 6. Conclusions

ILs-contained ionogels and PILs have showed combined characteristics of both ILs and the solid matrix, which implying many applications in various fields. According to the ion motion induced dielectric properties in ionogels and PILs, they have been applied as ER materials. Upon the review of the synthesis, ER and dielectric response of both inorganic ionogels and oPILs, it indicated that ILs which are physically or chemically restricted either in the matrix or on the surface of the matrix contribute to the interfacial polarization of the whole particle and then enhance the ER effect of the system. PILs received intensive study due to their accessible hydrophobic nature and the structure diversity in cations and anions both of which have significant effect on the ER response of PILs. The main issue in PILs-based ER materials is the narrow operating temperature caused by the low glass transition temperature of PILs. By introducing inorganic particles or semiconducting polymer to PILs, the ion motion in PILs is reduced, which successfully limits the electric leakage of the ER fluid. The same effect was found in the cross-linked PILs. For the lower temperature limit, anionic PILs with proper counter cations showed much excellent ER effect at 0 °C. On the side of inorganic ionogels, ILs have been widely used in synthesizing inorganics, however, which are not effectively used as electroresponsive ER materials. Therefore, there is still much work to do to understand the ER properties of inorganic ionogels. To avoid large leakage current and broaden the operating temperature are both challenges on the way to real application of ER fluids. Furthermore, long term stability of this kind of ER materials should be also concerned for their application in electric field controlled devices.

## Figures and Tables

**Table 1:** Dielectric characteristics of the suspensions of P[MTMA][TFSI] and SiO_2_@P[MTMA][TFSI] particles at room temperature (*φ* = 12 vol%), reprinted with permission from [95]. Copyright ACS, 2018.

Sample	$\varepsilon_{0}^{\prime }$	$\varepsilon_{\infty }^{\prime }$	$\Delta \varepsilon^{\prime }$	$\varepsilon^{\prime \prime a}$	λ(s)	$\sigma \left(Sm^{-1}\right)$	$\sigma_{p}^{b}\left(Sm^{-1}\right)$
P[MTMA][TFSI]	5.42	3.13	2.29	0.67	8.80 × 10^−5^	~7.08 × 10^−10^	~2.7 × 10^−8^
SiO_2_@P[MTMA][TFSI]	7.66	3.11	4.55	1.13	5.10 × 10^−3^	~2.66 × 10^−12^	~2.3 × 10^−9^

^a^ The value of dielectric loss factor at the relaxation peak. ^b^ The approximate dc conductivity of the particles calculated using Equation (1).
